# Development of Recombinant Human Collagen-Based Porous Scaffolds for Skin Tissue Engineering: Enhanced Mechanical Strength and Biocompatibility

**DOI:** 10.3390/polym17030303

**Published:** 2025-01-23

**Authors:** Yang Yang, Ting Yu, Mengdan Tao, Yong Wang, Xinying Yao, Chenkai Zhu, Fengxue Xin, Min Jiang

**Affiliations:** 1 State Key Laboratory of Materials-Oriented Chemical Engineering, College of Biotechnology and Pharmaceutical Engineering, Nanjing Tech University, Nanjing 211800, China; 2 Ningbo Institute of Technology, Beihang University, 399 Kangda Road, Ningbo 315832, China

**Keywords:** recombinant human collagen, porous scaffolds, mechanical properties, skin tissue engineering

## Abstract

Skin tissue engineering scaffolds should possess key properties such as porosity, degradability, durability, and biocompatibility to effectively facilitate skin cell adhesion and growth. In this study, recombinant human collagen (RHC) was used to fabricate porous scaffolds via freeze-drying, offering an alternative to animal-derived collagen where bovine collagen (BC)-based scaffolds were also prepared for comparison. The internal morphology of the RHC scaffolds were characterized by scanning electron microscopy (SEM) and the pore size ranged from 68.39 to 117.52 µm. The results from compression and fatigue tests showed that the mechanical strength and durability of RHC scaffolds could be tailored by adjusting the RHC concentration, and the maximum compressive modulus reached to 0.003 MPa, which is comparable to that of BC scaffolds. The degradation test illustrated that the RHC scaffolds had a slower degradation rate compared to BC scaffolds. Finally, the biocompatibilities of the porous scaffolds were studied by seeding and culturing the human foreskin fibroblasts (HFFs) and human umbilical vein endothelial cells (HUVECs) in samples. The fluorescent images and Cell Counting Kit-8 (CCK-8) assay revealed RHC porous scaffolds were non-cytotoxic and supported the attachment as well as the proliferation of the seeded cells. Overall, the results demonstrated that RHC-based scaffolds exhibited adequate mechanical strength, ideal biodegradability, and exceptional biocompatibility, making them highly suitable for skin-tissue-engineering applications.

## 1. Introduction

As the body’s primary barrier against external damage, the skin performs several crucial functions, including maintaining hydration, detecting environmental stimuli, and shielding against ultraviolet radiation [1]. The human skin consists of three main layers: the subcutaneous tissue, dermis, and epidermis [2]. The dermis is predominantly composed of skin fibroblasts, while the underlying layer primarily consists of vascular endothelial cells. Fibroblasts are responsible for synthesizing and secreting substantial amounts of collagen and elastin [3,4]. These synthesized collagen fibers form resilient networks through molecular interactions and crosslinking [5]. Providing both structural support and elasticity to the skin, enabling it to withstand external mechanical stress [6].

Skin wound repair is a dynamic, multifaceted process that occurs in stages [7]. However, in certain instances, particularly in cases of extensive acute wounds, chronic ulcers, diabetic wounds, or burns that resist healing, the skin’s intrinsic regenerative capacity is insufficient [8]. This has led to the development of tissue-engineered skin scaffolds, which offer promising solutions for facilitating repair and regeneration in such cases. For scaffolds intended for wound healing, several critical properties must be met, including excellent biocompatibility, suitable biodegradability, sufficient mechanical strength, and the ability to promote both vascularization and epithelialization [9]. The core technology of tissue engineering in this context lies in the careful selection of biocompatible materials, which are then utilized to fabricate scaffolds that support the attachment, proliferation, and differentiation of skin cells, thereby promoting the regeneration of functional skin tissue [10].

Materials commonly employed in the fabrication of tissue-engineered porous scaffolds predominantly consist of synthetic polymers, including polylactic acid (PLA), polylactic-co-glycolic acid (PLGA), polyvinyl alcohol (PVA), polyethylene oxide (PEO), polyethylene glycol (PEG), and polycaprolactone (PCL) [11]. However, these synthetic materials exhibit several limitations, such as insufficient biological activity, suboptimal cell–scaffold interactions, the use of toxic solvents during scaffold preparation, and an inability to accurately replicate the composition of the extracellular matrix (ECM) [12]. In contrast, collagen, a naturally derived material, offers notable advantages in terms of biocompatibility and biodegradability, facilitating enhanced cell attachment and proliferation [13]. As a bioactive substance, collagen plays a critical role in regulating cellular behavior, thus promoting the natural repair of tissues [14,15]. It influences intracellular signaling and cellular activities through various cell–material interaction mechanisms, including morphogenesis, ECM deposition, and tissue remodeling [16,17,18]. Furthermore, collagen is a principal component of skin tissue and is the most abundant and widely distributed structural protein in mammals. It is essential for maintaining the structural and biological integrity of the ECM, providing critical mechanical support to tissues [19].

The animal source collagen is commonly used as a raw biomaterial, is primarily sourced from the skin or tendons of cows and pigs, or from fish scales. However, this material exhibits batch-to-batch variability in quality. Additionally, animal-derived collagen may induce immunogenic reactions and poses a potential risk of transmitting animal viruses and prions [20]. For instance, studies have shown that scaffolds prepared from bovine collagen brings potential application dangers [21]. To overcome these limitations, there is an increasing focus on developing collagen alternatives that offer enhanced safety and efficacy for the preparation of tissue-engineered skin scaffolds [22].

Recombinant human collagen (RHC) is produced through genetic-engineering techniques and closely mimics the amino acid sequence of native-human collagen. In comparison to animal-derived collagen, RHC is preferred due to its enhanced safety profile, identical molecular weight, and high purity, making it an ideal candidate for use in tissue engineering applications [23]. Although RHC has emerged as a promising novel biomaterial in the field of biomedicine, the body of literature remains limited. To date, studies have primarily focused on RHC-based tissue-engineered skin substitutes and hydrogels, which have shown potential in promoting skin-defect repair and regeneration [24,25]. These preliminary studies have demonstrated the feasibility of utilizing RHC as a biomaterial in skin-tissue engineering. However, there is a paucity of comparative analyses between animal-derived collagen and RHC, particularly with regard to determining which type of collagen is more suitable for the fabrication of tissue-engineered porous scaffolds that enhance tissue regeneration and skin repair.

In order to investigate the feasibility of type-I RHC as biomaterial for skin tissue engineering, the tissue-engineered porous scaffolds were fabricated with varied RHC concentrations and compared with BC scaffold. The internal structure of the scaffolds was observed, and mechanical properties were thoroughly assessed through compression and fatigue testing. Furthermore, the in vitro cytocompatibility of the scaffold eluents was evaluated according to ISO-10993 standards. Proliferation and migration of seeded human foreskin fibroblasts (HFFs) and human umbilical vein endothelial cells (HUVECs) were assessed using cellular-viability assays and fluorescent staining, respectively. The final outcomes from this study confirmed that RHC with a porous structure could be a platform for cell attachment, migration and had a better capability for cell proliferation when compared to BC scaffold, which was attributed to the nature of RHC being close to that of human collagen. As such, RHC scaffolds could exhibit significant potential for applications in skin tissue engineering.

## 2. Materials and Methods

### 2.1. Preparation of Collagen Porous Scaffolds

**Figure 1:**
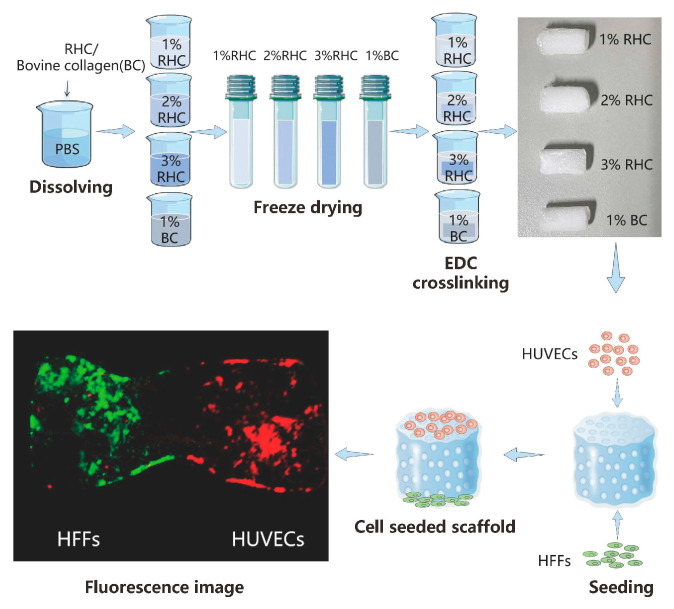
Schematic diagram for designed collagen scaffolds.

The process for fabricating the porous collagen scaffolds is schematically depicted in Figure 1. In brief, type-I recombinant human collagen (RHC, JLand Biotech Co., Ltd., Zhuji, China) and bovine collagen were used to prepare porous scaffold samples at varying concentrations (1%, 2%, and 3% *w*/*v*), as outlined in Table 1 and illustrated in Figure 1. As Figure. 1 illustrates, freeze-dried type-I RHC powder was dissolved in phosphate-buffered saline (PBS, Wisent, QC, Canada) solution to prepare a 1% (*w*/*v*) RHC solution. The solution was then poured into mold and frozen at −20 °C overnight. The frozen samples were subsequently subjected to freeze-drying using a freeze-dryer (LGJ-10D, Beijing, China) for 48 h to obtain the dry samples. A modified crosslinking method described from a previous study was used to reinforce the porous scaffolds [26]. Briefly, the samples were fully immersed in a 0.5% (*w*/*v*) solution of 1-ethyl-3-(3-dimethylaminopropyl) carbodiimide (EDC, Sinopharm Chemical Reagents Co., Ltd., Shanghai, China) for 10 h. Following crosslinking, the samples were washed three times with a PBS solution to remove any residual crosslinker. Porous scaffolds with 2% and 3% (*w*/*v*) RHC concentrations were prepared in the same manner, and the corresponding groups are listed in Table 1. For comparison, 1 g of lyophilized bovine collagen (BC) powder (kindly provided by the Ningbo Institute of Technology, Beihang University) was dissolved in 2% (*w*/*v*) acetic acid (Sinopharm Chemical Reagents Co., Ltd., Shanghai, China), and the pH was adjusted to 6.5 using 1 M sodium hydroxide (Sinopharm Chemical Reagents Co., Ltd., Shanghai, China), resulting in a 1% (*w*/*v*) BC solution. The BC porous scaffold was then fabricated following the same freeze-drying method as for the RHC scaffolds, followed by crosslinking in a 0.5% (*w*/*v*) EDC solution for 10 h and being rinsed three times with a PBS solution.

### 2.2. Fourier Transform Infrared Spectroscopy (FTIR)

The FTIR (Fourier Transform Infrared) spectrum of the samples was measured using a spectrometer (NICOLET iS5, ATR iD7, Thermo Scientific, Waltham, MA, USA). Vacuum dried scaffold was ground into a fine powder first, then placed on testing stage. The spectrum was recorded in the absorption mode with a scan step length of 2 cm^−^^1^, and the wavelength ranged from 4000 to 500 cm^−^^1^.

### 2.3. Morphological Properties

The internal structure of the porous scaffolds was observed using a scanning electron microscope. The freeze-dried samples were carefully sectioned using a scalpel and sputter-coated with gold using a turbomolecular pumped coater (Q150T, Quorum, East Sussex, UK). Finally, the internal morphologies of the scaffolds were observed using an electron scanning microscope (Regulus 8230, Hitachi, Tokyo, Japan) at an acceleration voltage of 3 kV.

### 2.4. Mechanical Properties Testing

The mechanical properties of the samples were assessed using a universal testing machine (UTM4304X, Suns, Shenzhen, China) in the uniaxial compression mode. Briefly, each sample, with a thickness of 5 mm and a diameter of 10 mm, was compressed at a constant loading rate of 2 mm/min, and the stress at 70% strain was recorded. The compression modulus was derived from the initial linear region of the stress–strain curve. Fatigue testing was also performed using the same universal testing machine. The samples underwent repeated compression cycles at a constant rate of 10 mm/min, with strains ranging from 0% to 50% and back to 0% over 20 cycles.

### 2.5. In Vitro Degradation

The biodegradation rate of the sample was evaluated using a gravimetric method. Initially, the freeze-dried scaffold was weighed to determine its initial mass, denoted as W1. The scaffold was then immersed in 5 mL of PBS for a 14-day period, with the PBS solution being replaced every 2 days. At designated time points (1, 3, 7, and 14 days), the scaffold was retrieved, rinsed three times with distilled water to remove any residual PBS, then freeze-dried, and weighed again, with the post-degradation mass recorded as W2. The biodegradation rate was calculated using the following formula: (W1 − W2)/W1 × 100%, where W1 represents the initial mass of the scaffold and W2 is the mass of the scaffold at each time point during the degradation process.

### 2.6. Biocompatibility

Fibroblasts and endothelial cells are the principal cells in human skin tissue. In this study, HFFs and HUVECs were selected to evaluate the biocompatibilities of the collagen scaffolds. Both cell lines were purchased from Zhong Qiao Xin Zhou Biotechnology Co., Ltd. (Shanghai, China). Complete Dulbecco’s modified Eagle’s medium with 10% fetal bovine serum, 2% HEPES buffer, 2% penicillin/streptomycin, 1% L-glutamine, 1% non-essential amino acids, and 0.85 mM ascorbic acid (Gibco Invitrogen, New York, NY, USA) was used to culture HFFs. F-12k medium with 10% FBS, 2% penicillin/streptomycin, 1% L-glutamine, and 1% non-essential amino acids (Gibco Invitrogen, New York, NY, USA) was used to culture HUVECs. All cells were cultured in a 5% CO_2_ humidified incubator at 37 °C.

#### 2.6.1. Cytotoxicity Test

The cytotoxicity assessment of RHC and BC scaffolds was conducted in accordance with ISO-10993 standards by evaluating the elution of samples. Initially, the ultraviolet-sterilized scaffolds were placed in a 12-well culture plate. Following this, 2.5 mL of fresh medium was added to each well to fully immerse the samples. The samples were then incubated at 37 °C for 24 h, after which the eluents were collected. HFFs were seeded into a 96-well plate at a density of 3000 cells per well and incubated for 24 h to achieve confluence. Subsequently, the medium in each well was replaced with 100 μL of the sample eluents and cultured for an additional 24 h. Cells cultured in fresh medium served as the control group. After 24 h, 10 μL of Cell Counting Kit-8 (CCK-8, Biyuntian, Shanghai, China) solution was added to each well, and the plates were incubated at 37 °C for 3 h. The absorbance was measured at a wavelength of 450 nm using a microplate reader (Infinite M200 PRO, Tecan, Vienna, Austria), and the results were expressed as the relative viability compared to the control group. The morphology and condition of HFFs cultured in the extracted eluents were evaluated using calcein-AM/PI staining at specified time points (24 h). Fluorescent images were captured using an inverted fluorescence microscope (X-81, Olympus, Tokyo, Japan). The cytotoxicity of the scaffold eluents towards HUVECs was assessed using the same methodology.

#### 2.6.2. In Vitro Culturing

To assess the suitability of the scaffold for supporting the growth and proliferation of skin cells, in vitro experiments were conducted in which HFFs and HUVECs were seeded and cultured within the porous scaffolds. Cell viability was further evaluated using the CCK-8 assay. Briefly, scaffold samples with dimensions of 1 mm × 1 mm × 3 mm were sterilized under ultraviolet (UV) light for 40 min. HFFs were then seeded onto the scaffolds at a density of 15,000 cells per scaffold, and the scaffolds were placed in culture medium and incubated at 37 °C with 5% CO_2_. The culture medium was refreshed every two days. HUVECs were seeded and cultured using the same procedure. On days 3 and 7 of culture, 10 μL of CCK-8 reagent was added to each well, and the samples were incubated for an additional 3 h. After incubation, 100 μL of the reacted medium from each sample was transferred to a 96-well plate, and absorbance was measured at 450 nm using a microplate reader to determine cell viability. The distribution of HFFs and HUVECs cultured on the RHC and BC scaffolds was assessed through fluorescence staining using ER-Tracker (Yeasen, Shanghai, China) at designated time points (days 3 and 7). Fluorescent images were captured using the inverted fluorescence microscope.

### 2.7. Statistical Analyses

In this study, three replicates were involved in each set of test (n = 3), and the data were expressed as mean ± standard deviation (SD). Origin Pro 2021 was used for statistical analysis, and the Tukey test was used to detect significant differences. * *p* ≤ 0.05, ** *p* ≤ 0.01, and *** *p* ≤ 0.001 were all significant differences, and *p* > 0.05 was considered not statistically significant.

## 3. Results and Discussion

### 3.1. FTIR Spectroscopy

**Figure 2:**
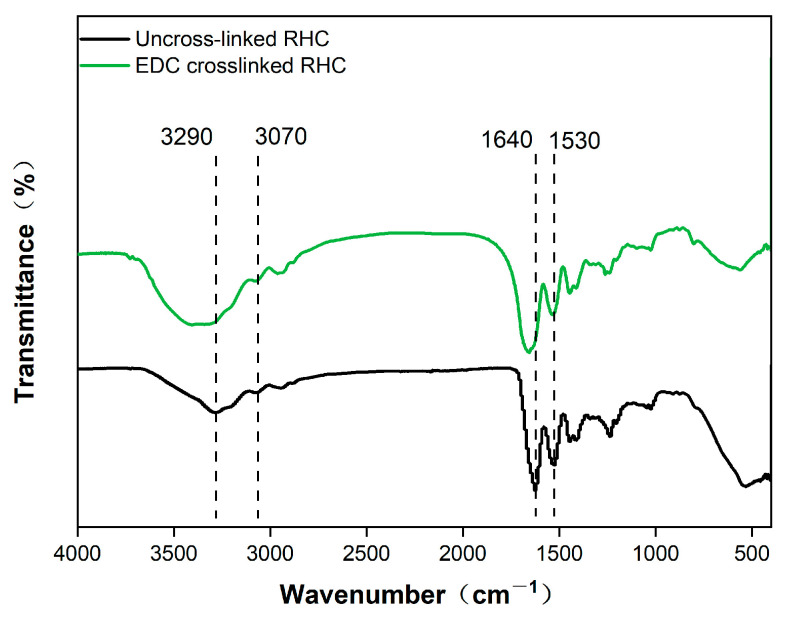
Fourier infrared spectroscopy analysis of RHC scaffolds with or without EDC crosslinking.

The FTIR spectra of the RHC scaffold, both with and without EDC crosslinking, are presented in Figure 2. In the hydrogen bond region (4000–2500 cm^−^^1^), an amide A band is observed at 3290 cm^−^^1^, corresponding to the interaction between N-H stretching vibrations and hydrogen bonding. An amide B band appears at 3070 cm^−^^1^, attributed to the asymmetric and symmetric stretching vibrations of the -CH_2_ group [27]. In the region associated with double bonds (2000–1500 cm^−^^1^), a peak at 1640 cm^−^^1^ is identified as amide I, resulting from the C=O stretching vibration [28]. A peak at 1530 cm^−^^1^ corresponds to amide II, which is associated with δN-H deformation and νC-N stretching vibrations. The FTIR spectra of both uncrosslinked and EDC-crosslinked RHC scaffolds indicate a significant enhancement in the vibratory intensity of N-H related functional groups, particularly those corresponding to amides A and I, indicating the formation of new iso-peptides between RHC molecules [29].

### 3.2. Internal Morphology

**Figure 3:**
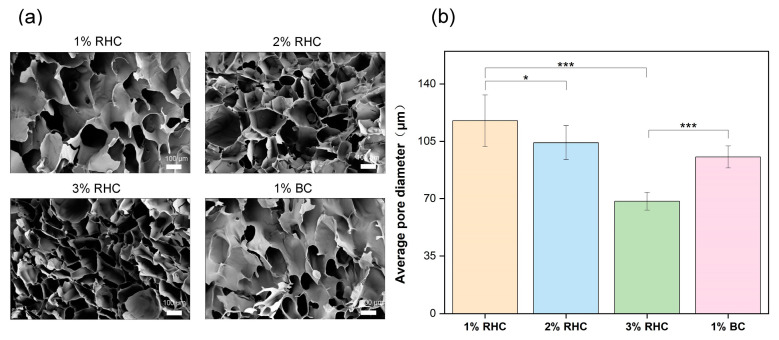
The internal morphology of scaffolds. (**a**) Pore structure of the freeze-dried sample, scale bar = 100 μm; (**b**) the average pore size of the scaffolds. Significance is indicated with * *p* ≤ 0.05 and *** *p* ≤ 0.001.

In addition to the biocompatibility of the porous scaffolds, it is crucial to consider their internal morphology, including pore size and structural configuration [30]. These parameters not only influence cell–scaffold interactions, such as cell adhesion, migration, and proliferation [31], but also manipulate the scaffold mechanical properties. Previous studies have demonstrated that scaffolds with larger pore sizes and thinner walls typically exhibit reduced mechanical strength [32]. The morphology of the scaffolds was characterized using scanning electron microscopy (SEM), with the results presented in Figure 3. As illustrated in Figure 3a, all scaffold samples exhibit a highly porous structure with relatively uniform pore distribution. Pore size was found to be directly proportional to the concentration of RHC, with the 3% RHC scaffold exhibiting the smallest pore size, approximately 68.39 µm (Figure 3b). This observation aligns with previous research, which established a negative correlation between polymer concentration and both pore size and porosity in the fabrication of porous scaffolds [33]. This phenomenon can be attributed to the increased polymer concentration, which inhibits water crystallization during the freezing process, thereby resulting in smaller pore sizes [34].

### 3.3. Mechanical Properties

**Figure 4:**
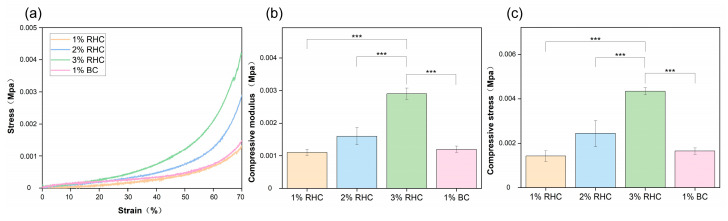
Mechanical properties of the samples. (**a**) Stress–strain curves of the RHC and BC scaffolds; (**b**) compressive modulus of the RHC and BC scaffolds; and (**c**) compressive stress of the RHC and BC scaffolds. Significance is indicated with *** *p* ≤ 0.001.

The collagen scaffolds intended for tissue engineering must be capable of withstanding specific mechanical loads to ensure their stability during the practical application of skin wound treatment [35]. In this study, the mechanical properties of the porous scaffolds were evaluated through compressive-strength testing and fatigue analysis. As shown in Figure 4a, the stress–strain curve of the RHC scaffold follows a characteristic pattern, initially linear and subsequently nonlinear, indicating that the scaffold exhibits viscoelastic behavior akin to that of human tissues [36]. As shown in Figure 4b, the RHC concentration is directly proportional to the compression modulus of the scaffolds. The compression modulus of the 3% RHC scaffold increased significantly when compared to 1% RHC scaffold which is showing the lowest value.

As depicted in Figure 4c, the trend in the maximum stress values of the scaffolds across the four groups was consistent with the observations in Figure 4b. The average maximum stress values for the RHC scaffolds were 0.0014, 0.0025, and 0.0044 MPa, respectively. Notably, compressive stress was found to be directly proportional to the RHC concentration. Significant differences were observed between the 3% RHC scaffold and the 1% BC scaffold. Both the compression modulus and the compressive stress at 70% deformation further confirmed that the 3% RHC scaffolds exhibited superior mechanical properties compared to the other groups. These findings are in agreement with previous studies on porous scaffolds derived from animal collagen [21,37].

**Figure 5:**
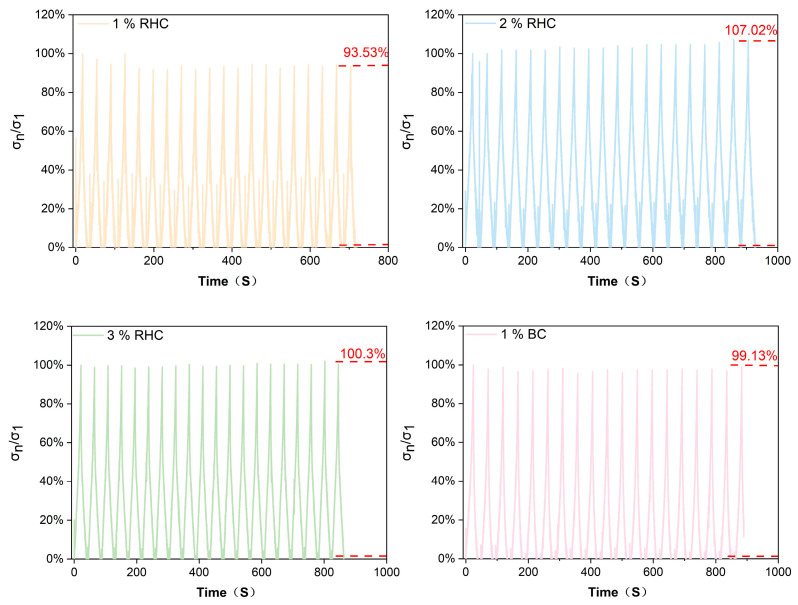
The compressive stress ratio of σ_n_/σ_1_ of four scaffolds as a function of time under 20 cycles.

Compression fatigue testing of tissue-engineered skin scaffolds is mainly conducted to evaluate their long-term stability and durability under mimicked physiological conditions. The mechanical fatigue test can reflect the anti-fatigue strength and deformation ability of the scaffold during usage in vivo [38,39]. As Figure 5 illustrates, to evaluate the fatigue resistance of the RHC scaffolds, the ratio of compressive stress (σ_n_/σ_1_) was recorded for each scaffold over 20 cycles of compression within 1000 s. Here, σ_n_/σ_1_ represents the ratio of the maximum stress in each cycle (σ_n_) to the maximum stress of the first cycle (σ_1_). The peak σ_n_/σ_1_ values for each sample remained relatively stable after 20 cycles of repeated compression. At the conclusion of the cyclic compression, as the RHC concentration increased from 1% (*w*/*v*) to 3% (*w*/*v*), the peak σ_n_/σ_1_ value was increased from 93.53% to 107.2% and then decreased to 100.3%. This increase behavior indicated that the higher RHC concentrations could enhance the scaffold’s recoverable capacity. In addition, another study investigated the compressive behavior of collagen scaffold and confirmed that the cycle-compression applied on the collagen scaffold could result in further orientation of the molecular chain with denser structure and better compressive resistance [40]. In addition, with a continuous addition of collagen into the scaffold to be 3% RHC, the elasticity for the scaffold structure could be reduced with better rigidity [41]. Consequently, the fatigue compression performance of the scaffold is directly correlated with its overall durability [42,43]. Therefore, analyzing the compressive behavior of the scaffold under specific loading conditions provides valuable insights into its anti-deformation capacity and lifespan, offering essential guidance for the design and application of scaffolds.

### 3.4. In Vitro Degradation

**Figure 6:**
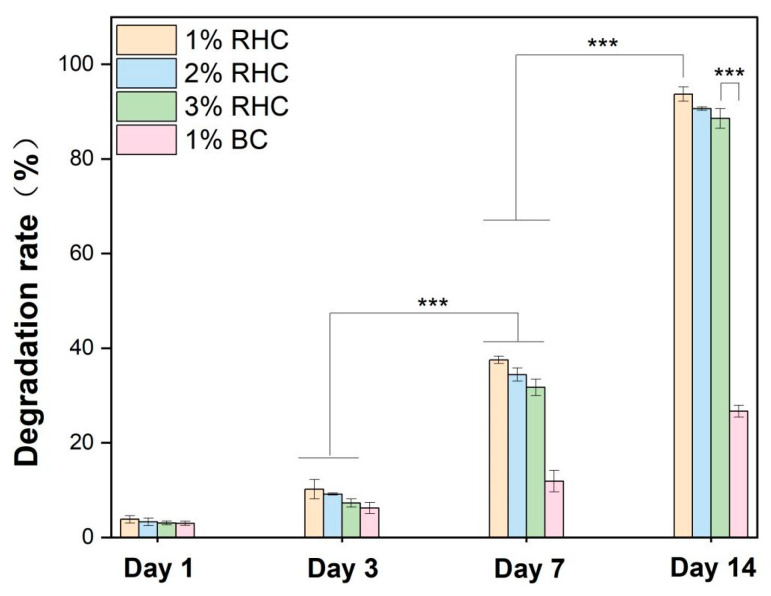
Degradation rates of the RHC and BC scaffolds at selected time points. Significance is indicated with *** *p* ≤ 0.001.

An appropriate biodegradation rate is a critical consideration in the design of tissue-engineered skin scaffolds. The rate of degradation must be aligned with the rate of new tissue growth to ensure effective scaffold performance [44]. Biodegradable collagen scaffolds are required to support the proliferation, migration, and growth of skin cells, ultimately degrading in a controlled manner, leaving behind healthy, regenerated tissue [45]. The degradation results are presented in Figure 6. As time progressed, the degradation rates increased progressively. On day 7, significant differences were observed between the RHC and BC scaffolds, with the degradation rate of all RHC samples surpassing that of the BC samples. This trend persisted until day 14. By the end of the study, at least 86% of the RHC scaffolds had degraded, while only 26% of the bovine collagen scaffolds had undergone degradation. Additionally, the degradation rate was found to be concentration-dependent, with the 3% RHC scaffold exhibiting the slowest degradation. This can be attributed to the denser three-dimensional internal structure formed at higher concentrations, which confers greater structural integrity and mechanical strength, thereby maintaining scaffold stability and reducing the rate of degradation [46]. Furthermore, the crosslinking density of the scaffolds was found to influence their biodegradation rate, with higher crosslinking contributing to slower degradation in chemically reinforced scaffolds [47]. Notably, the biodegradation rate of the 3% RHC scaffold aligns with the tissue regeneration capacity observed in previous studies [48].

### 3.5. Biocompatibility

#### 3.5.1. Cytotoxicity Test

**Figure 7:**
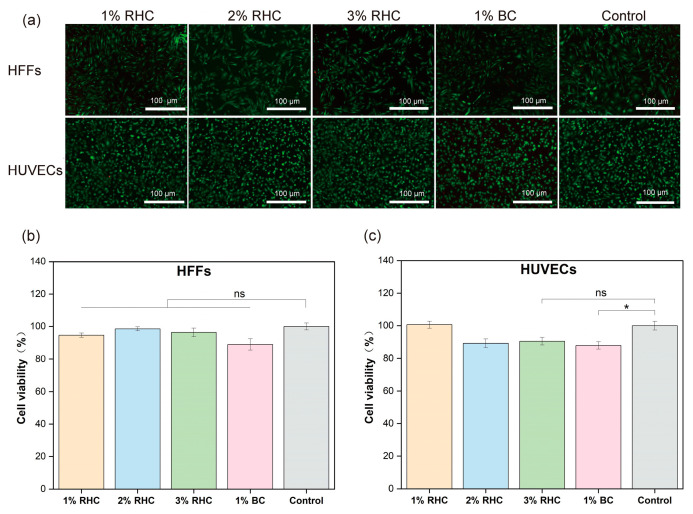
Cytotoxicity test of the samples. (**a**) Live/dead fluorescence staining of HFFs and HUVECs cells, scale bar = 100 μm; (**b**) the viability of HFFs; and (**c**) the viability of HUVECs. Significance is indicated with * *p* ≤ 0.05, and not statistically significant is indicated with ns.

For tissue-engineered scaffolds, it is essential that the material is non-toxic and provides a stable platform conducive to cell adhesion and proliferation [49]. Cytotoxicity testing is crucial for assessing the suitability of materials as scaffolds for tissue engineering applications [50]. To evaluate the cytotoxicity of the porous scaffolds, a CCK-8 assay was employed to assess the viability of HFFs and HUVECs cultured in scaffold extracts. Additionally, cell morphology was examined via fluorescence staining. As shown in Figure 7a, both HFFs and HUVECs displayed healthy growth, with minimal, red-stained dead cells, indicating low cytotoxicity. HFFs exhibited a characteristic elongated, spindle-like shape with irregular edges, while HUVECs showed a more rounded, pebble-like appearance and a uniform size. Figure 7b demonstrates that the cell viability of both HFFs and HUVECs did not differ significantly between the tested samples and the control group. In Figure 7c, it is evident that HUVECs cultured in the 1% RHC extract exhibited the highest viability, with the 3% RHC group showing no significant difference compared to the control. In contrast, the BC scaffold demonstrated a significant reduction in cell viability relative to the control group (*p* < 0.05). These results confirm that neither RHC nor BC scaffolds were cytotoxic to the selected cell types, in accordance with ISO-10993 standards, which stipulate that a material is considered non-cytotoxic if cell viability exceeds 70% of the control group. However, the BC scaffold displayed the lowest cell viability among all tested samples, highlighting RHC as a superior choice, especially considering that no organic solvents were involved in the preparation of the RHC porous scaffolds [51].

#### 3.5.2. In Vitro Test

**Figure 8:**
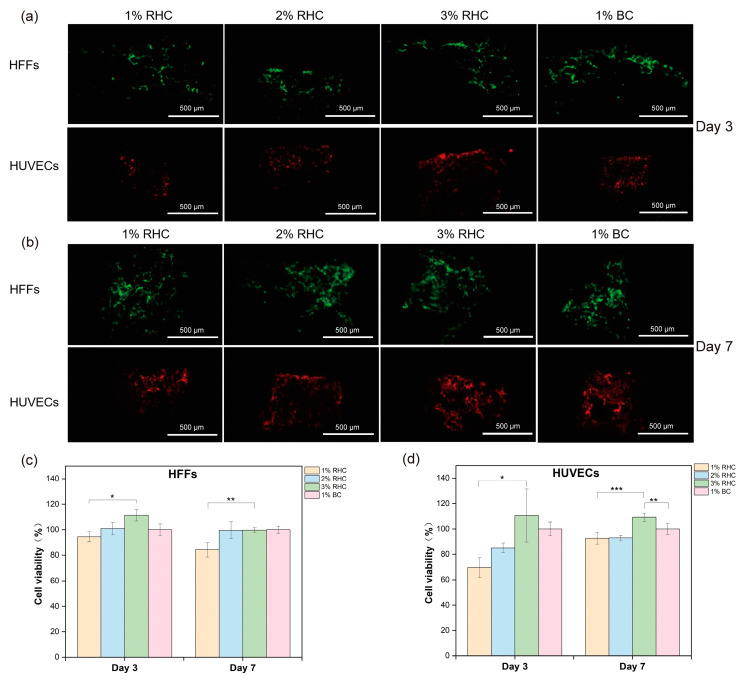
In vitro test of cell seeded scaffolds. (**a**) Fluorescence staining of HFFs (green) and HUVECs (red) within scaffolds at day 3, scale bar = 500 μm; (**b**) fluorescent staining of cell proliferation of HFFs and HUVECs within scaffolds at day 7, scale bar = 500 μm; (**c**) viabilities of HFFs; and (**d**) viabilities of HUVECs. Significance is indicated with * *p* ≤ 0.05, ** *p* ≤ 0.01, and *** *p* ≤ 0.001.

Collagen, as a key structural protein of the extracellular matrix (ECM), plays a critical role in maintaining tissue structural integrity and biological function due to its natural origin and distinct three-dimensional configuration [52]. Directly inoculating cells onto scaffolds mimics the implantation process and allows for the examination of cell–material interactions during application [53]. In this study, cell proliferation within the scaffolds was assessed using fluorescent imaging and cell viability tests on days 3 and 7, respectively. As shown in Figure 8a,b, the number of cells increased over time, with a higher cell count observed on the BC and 3% RHC scaffolds compared to the 1% RHC scaffold. These trends are consistent with the CCK-8 assay results, which indicated slightly higher cellular activity in both the 3% RHC and BC scaffolds compared to the 1% RHC group at the selected time points. Furthermore, HUVEC viability was significantly higher in the 3% RHC samples than in the BC samples. Collectively, these findings suggest that scaffolds fabricated from higher RHC concentrations promote more favorable conditions for skin cell growth, whereas lower RHC concentrations support cell proliferation in a more gradual but sustained manner [54]. This observed behavior may be attributed to the pore size and mechanical strength of the scaffolds, as previous studies have indicated that scaffolds with smaller pores are more conducive to cell attachment, growth, and proliferation [55].

## 4. Conclusions

In order to evaluate potential capability for skin-tissue-engineering applications, RHC scaffolds were designed as a concentration from 1% to 3% (*w*/*v*) and compared with a BC scaffold. The scaffolds developed exhibited favorable mechanical performance, such as significant improvement on compressive strength and fatigue resistance with an increase in RHC concentrations. In addition, the biodegradation behavior of scaffold confirmed that 3% RHC scaffold displayed the slowest degradation rate, which was ideal for tissue regeneration. Importantly, in vitro studies further confirmed the biocompatibility of the RHC scaffolds where higher RHC concentrations promote better cell growth and cell attachment. When compared to a BC scaffold, the potential of a 3% RHC scaffold as a promising material for skin regeneration is notable, with it offering an optimal balance of mechanical stability, biodegradability, and biocompatibility, making it a promising candidate for tissue engineering applications.

## Figures and Tables

**Table 1:** Types of fabricated scaffolds.

Scaffolds	Concentration (*w*/*v*)
RHC	1%
RHC	2%
RHC	3%
BC	1%

## Data Availability

The original contributions presented in the study are included in the article, further inquiries can be directed to the corresponding author.
